# Characterization and chromosomal mapping of the *Dgmar*MITE transposon in populations of *Dichotomius* (*Luederwaldtinia*) *sericeus* species complex (Coleoptera: Scarabaeidae)

**DOI:** 10.1590/1678-4685-GMB-2017-0230

**Published:** 2018-06-04

**Authors:** Igor Costa Amorim, Rafaelle Grazielle Coelho Costa, Crislaine Xavier, Rita de Cássia de Moura

**Affiliations:** 1 Universidade de Pernambuco Universidade de Pernambuco Instituto de Ciências Biológicas Laboratório de Biodiversidade e Genética de Insetos RecifePE Brazil Laboratório de Biodiversidade e Genética de Insetos, Instituto de Ciências Biológicas, Universidade de Pernambuco, Recife, PE, Brazil; 2 Universidade Federal de Pernambuco Universidade Federal de Pernambuco Centro de Biociências Departamento de Genética RecifePE Brazil Departamento de Genética, Centro de Biociências, Universidade Federal de Pernambuco, Recife, PE, Brazil

**Keywords:** Mariner-like elements, cross-mobilization, chromosome evolution

## Abstract

Transposable elements are dispersed repetitive DNA sequences that can move within the genome and are related to genome and chromosome evolution, adaptation, and speciation. The aim of this study was to characterize and determine the chromosomal location and accumulation of a *Mariner*-like element in populations of four phylogenetically related species of the *Dichotomius* (*Luederwaldtinia*) *sericeus* complex. Mapping of the isolated element was performed by fluorescent *in situ* hybridization in different populations of analyzed species. Characterization of the isolated element revealed a degenerated transposon, named *Dgmar*MITE. This transposon is 496-bp-long, AT rich (57%), and contains 24 bp terminal inverted repeats. *In situ* mapping revealed presence of this element only in two out of four species analyzed. *Dgmar*MITE sites were located in heterochromatic and euchromatic regions and varied in location and number on the karyotypes of *Dichotomius* (*L.*) *gilletti* and *D.* (*L.*) *guaribensis* across different populations. These results demonstrate differential accumulation of the *Dgmar*MITE in genomes of these species, which is probably due to the occurrence of ectopic recombination and cross-mobilization of the element mediated by the transposase of closely related or unrelated transposable elements.

## Introduction

*Mariner* transposable elements are DNA transposons that exhibit broad diversity in their structure. *Mariner* elements are characterized by a size of about 1,300 bp, a single ORF (open reading frame) encoding a transposase, a conserved catalytic domain [DD(34)D] necessary for transposition, and two terminal inverted repeats (TIRs) of 28-30 bp flanked by a TA dinucleotide resulting from target site duplications (Robertson, 1995; Robertson and Lampe, 1995; Plasterk *et al.*, 1999). During transposition, the encoded transposase recognizes the TIRs and catalyzes excision of the two DNA strands at the donor site and fusion of the element at another site in the genome (Lampe *et al.*, 1996).

The *Mariner* elements are probably the most widely distributed family of transposable elements (TEs) in nature, being represented in diverse taxa, such as rotifers, fungi, plants and vertebrates. Their wide distribution in metazoan species, including arthropods (Robertson and Lampe, 1995; Wallau *et al.*, 2014), is probably related to horizontal transfer events (Robertson 1995; Robertson and Lampe, 1995; Lampe *et al.*, 2003) which, for example, account for the presence of the *Mariner*_Tbel and *Mariner*1_BT families in phylogenetically distant species such as insects and mammals (Oliveira *et al.*, 2012). These elements have been found in a wide range of insects from different orders, including Diptera, Hemiptera, Hymenoptera, Lepidoptera, Orthoptera and Coleoptera (Robertson and Lampe, 1995; Palacios-Gimenez *et al.*, 2014).

The existence of nonfunctional *Mariner* elements is common, including a large number of inactive copies in different genomes (Lohe *et al.*, 1995). Some of those inactive elements, the miniature inverted repeat transposable elements (MITEs), do not encode the enzyme necessary for their transposition and therefore require the transposase of other elements for their mobilization (Kidwell, 2005). The origin of these TEs is related to the internal degeneration of autonomous elements (Deprá *et al.*, 2012). MITEs are distinguished from their autonomous counterparts by their high copy number, compact structure, short terminal inverted repeats, genic preference, and DNA sequence identity (Feschotte *et al.*, 2002, Feng, 2003).

Regarding the speciose order Coleoptera, *Mariner* elements have so far been described in only a few species belonging to the families Chrysomelidae, Buprestidae, Cerambycidae, Laemophloeidae, Meloidae, Scarabaeidae, Staphylinidae and Tenebrionidae (Robertson, 1993; Robertson and Macleod, 1993; Robertson *et al.*, 2002; Lampe *et al.*, 2003; Richards *et al.*, 2008; Rivera-Vega and Mittapalli, 2010; Oliveira *et al.*, 2013; Xavier *et al.*, 2014). However, data from chromosome mapping of *Mariner* TEs in Coleoptera are limited to two species of *Coprophanaeus*, one of *Diabroctis* (Oliveira *et al.*, 2013) and one *Dichotomius* (Xavier *et al.*, 2014), all genera belonging to the family Scarabaeidae. Despite the small number of studies, TEs have been associated with important evolutionary processes in Scarabeidae, such as chromosome rearrangements (Oliveira *et al.*, 2013), dispersion of 18S rDNA sites (Cabral-de-Mello *et al.*, 2011a,b), and dynamics of the repetitive DNA fraction that composes the constitutive heterochromatin (CH) in the genomes of *Dichotomius* species (Cabral-de-Mello *et al.*, 2011c).

Cytogenetic studies have been carried out in only 18 of the 165 described *Dichotomius* species, including molecular cytogenetics studies in 15 species (Cabral-de-Mello *et al.*, 2008, 2011a,b; Silva *et al.*, 2009; Korasaki *et al.*, 2012; Xavier *et al.*, 2014). This genus presents groups of closely related species (Sarmiento-Garcés and Amat-Garcia, 2009), including *Dichotomius* (*Luederwaldtinia*) *sericeus* complex (Coleoptera: Scarabaeidae). This complex was recently taxonomically revised by Valois *et al.* (2017), raising the number of species from five to eight. More specifically, *D. sericeus var. aterrimus* (Luederwaldt, 1929) was synonymized with *D. sericeus* and four new taxa, *D. guaribensis*, *D. gilletti*, *D. iannuzziae*, and *D. catimbau* have been described.

Species of the genus *Dichotomius* present the derived karyotype 2*n* = 18, Xy_p_, with meta-submetacentric chromosome morphology and presence of a large metacentric pair (Silva *et al.*, 2009; Cabral-de-Mello *et al.*, 2011a). The constitutive heterochromatin, located in pericentromeric regions of all autosomes, show similar patterns of highly and moderately repeated DNAs (C0t-1 DNA fraction) distribution in the six analyzed species (Cabral-de-Mello *et al.*, 2011c). Furthermore, the 45S rDNA is predominantly located in the distal region of the third autosome pair, whereas the 5S rDNA and H3 histone were co-located in the proximal region of the second pair in 14 analyzed species (Cabral-de-Mello *et al.*, 2011a,b).

The aim of this study was to access whether distinct populations present differential patterns of location and accumulation of *Mariner*-like elements. Therefore, we characterized and mapped *Dgmar*MITE sequences in chromosomes of phylogenetically related species of the *Dichotomius* (*Luederwaldtinia*) *sericeus* complex belonging to different populations.

## Material and Methods

### Specimens sampling

All species investigated herein belong to *Dichotomius* (*L.*) *sericeus* complex. *Dichotomius* (*Luederwaldtinia*) *gilletti* and *D.* (*L.*) *iannuzziae* were collected in Aldeia (7º53’48’ S, 35º10’’47’W) and Igarassu (7°48’37’’S; 34°57’25’’W), remnants of the Atlantic Rain Forest in the state of Pernambuco, Brazil. Additionally, individuals of *D.* (*L.*) *schiffleri* and *D.* (*L.*) *guaribensis* were collected in Maracaípe (8°31’26”S 35°1’31”W), Pernambuco. *D.* (*L.*) *guaribensis* was also collected in REBIO Guaribas (6°42’41”S 35°11’17”W), Paraíba, Brazil. The individuals were collected using pitfall traps, in compliance with IBAMA/SISBIO guidelines (Permanent license No. 16278-1 for the collection of zoological material, authorization No. 41761-4 for collection in a Federal Conservation Unit for scientific purposes, and the license No. 50438-1, specific for *D.* (*L.*) *schiffleri*). The specimens were identified by the taxonomist Dr. Fernando Silva, from the Universidade Federal do Pará, in Brazil.

### DNA extraction and isolation of the transposable element

DNA samples of the four species of *Dichotomius* mentioned above were obtained from the pronotum tissue. Genomic DNA was extracted according to the protocol described by Sambrook and Russell (2001). *Mariner* elements were amplified by PCR using the MOS_N679 primer from *Drosophila* (5’GCCATATGTCGAGTT TCGTGCCA) (Zhang *et al.*, 2001).

The volume of each PCR assay was 25 μL containing 12 ng genomic DNA, 1x PCR buffer, 5 mM MgCl_2_, 0.2 mM dNTP (Invitrogen), 1 pmol primer, and 1 U *Taq* polymerase (Invitrogen). The PCR conditions were 94 ºC for 5 min, followed by 30 cycles at 94 ºC for 30 s, 49 ºC for 30 s and 72 ºC for 1.20 s, and a final extension step at 72 ºC for 5 min.

PCR products were separated by electrophoresis on 1% agarose gel. A band of approximately 500 bp obtained from *D.* (*L.*) *gilletti* (Supplementary Figure S1) was isolated from the gel using the Zymoclean™ Gel DNA Recovery Kit (Sinapse) according to the protocol of the manufacturer.

### Cloning and sequencing

The isolated DNA fragment was cloned using the pGEM-T Easy Vector (Promega) according to manufacturer’s instructions. The insert was isolated by PCR using the M13 primer (M13F 5’-GTAAAACGACGGC CAG/M13R 5’-CAGGAAACAGCATATGAC). Concentrations of PCR reagents were the same as those described above. The PCR conditions were 95 ºC for 3 min, followed by 30 cycles at 95 ºC for 30 s, 55 ºC for 1 min and 72 ºC for 2 min, and a final extension step at 72 ºC for 5 min. For sequencing, the M13 PCR product was purified with ExoSAP-IT (Affymetrix/USB) and sequenced in an ABI3730XL automated sequencer (Applied Biosystems) by Macrogen Inc.

### Editing and analysis of the transposable element

The chromatograms of forward and reverse strands of the M13 PCR product were analyzed with the Pregap4 software of the Staden package (Bonfield *et al.*, 1995) in order to generate consensus sequences. Only bases with a Phred value of 20 or higher were considered in this analysis. Vector sequences were removed using the VecScreen tool. The sequence obtained (accession number: KX787885) was used as a query in GenBank Blast and RepBase Censor for correct identification and classification of the elements. In addition, the presence of ORFs was investigated using the ORFfinder tool.

### Chromosome preparations, C-banding and fluorescent *in situ* hybridization (FISH)

Cytological preparations of our four target species were obtained by the classical testicular follicles squashing technique in 50% acetic acid. Two male individuals of each species were analyzed. C-banding was performed on *D.* (*L.*) *gilletti* and *D.* (*L.*) *guaribensis* karyotypes following Sumner (1972). FISH was performed according to the protocol of Pinkel *et al.* (1986), with modifications as proposed by Cabral-de-Mello *et al.* (2010). The probe of the transposable element was labeled with dUTP-digoxigenin (Roche) and detected with anti-digoxigenin-rhodamine (Roche).

### Photodocumentation

Hybridization images were captured with a Leica DM 2500 epifluorescence microscope. Brightness and contrast of the images were optimized using the Photoshop CS5 program.

## Results

**Figure 1:**
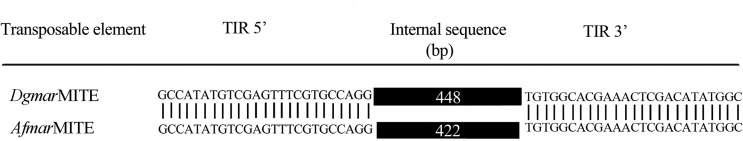
Alignment of terminal inverted repeats (TIRs) of the elements *Dgmar*MITE and *AfMar*2.

The presence of fragments amplified by the MOS_N679 primer of *Mariner* elements (Figure S1) and hybridization signals of the *DgmarMITE* were observed in only two out of the four analyzed species, namely *Dichotomius* (*L.*) *gilletti* and *D.* (*L.*) *guaribensis*. The element isolated from *D.* (*L.*) *gilletti* was 496 bp-long, rich in AT (57%), and had perfect TIRs of 24 bp. The consensus sequence used as a query sequence in GenBank and RepBase revealed 100% similarity with TIRs of the *AfMar*2 *Mariner*-like element of the grasshopper *Abracris flavolineata* (Figure 1) (accession number: KJ829354.1). The sequence between TIRs had no similarities to previously described elements. In addition, the largest identified ORF contained only 30 amino acids and showed no similarity to any transposase.

**Figure 2:**
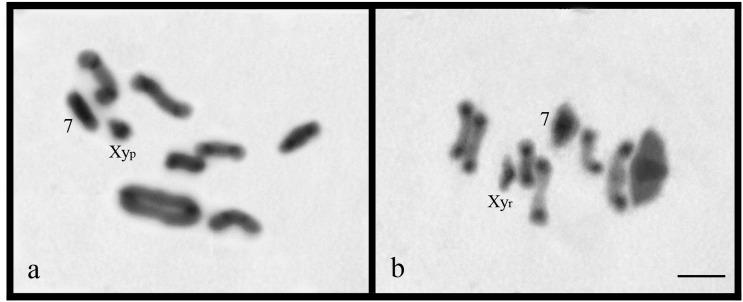
C-banding in metaphase I of *Dichotomius* (*L.*) *gilletti* (a) and metaphase I of *D.* (*L.*) *guaribensis* (b). Bar = 5 μm.

The species *Dichotomius* (*L.*) *guaribensis*, *D.* (*L.*) *gilletti*, *D.* (*L.*) *iannuzziae* and *D.* (*L.*) *schiffleri* presented similar karyotypes with 2*n* = 18, and meta-submetacentric chromosomal morphology. However, distinct sexual determination systems were obverved: *D.* (*L.*) *gilletti*, and *D.* (*L.*) *iannuzziae* had a Xy_p_ system, whereas *D.* (*L.*) *schiffleri* and *D.* (*L.*) *guaribensis* presented a Xy_r_ sex bivalent configuration (Figure 2). C-banding revealed pericentromeric constitutive heterochromatin in all autosomes, and additionally, along the entire length of the seventh bivalent and X chromosome of *Dichotomius* (*L.*) *gilletti* and *D.* (*L.*) *guaribensis* (Figure 2a,b).

**Figure 3:**
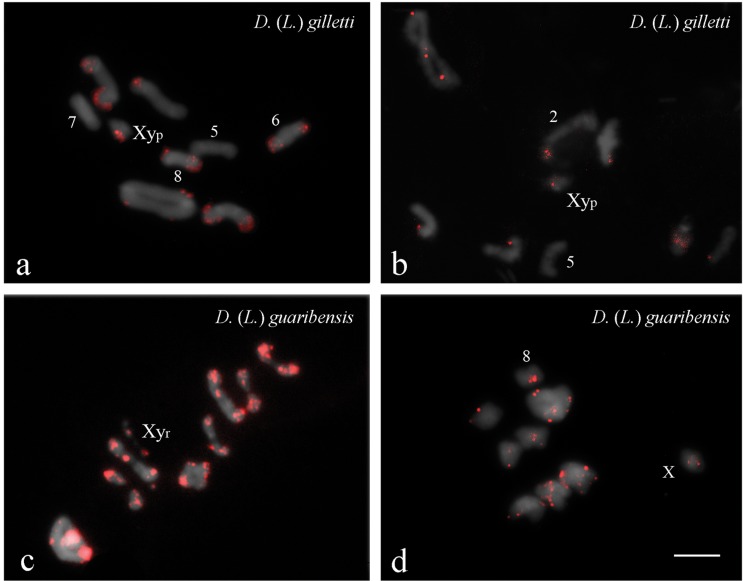
Fluorescent *in situ* hybridization of the element *Dgmar*MITE in meiotic cells of two individuals of *Dichotomius* (*Luederwaldtinia*) *gilletti* (a, b) and *D.* (*L.*) *guaribensis* (c, d). (a) Metaphase I of an individual from Igarassu population; (b) diplotene of specimen from Aldeia population; (c) metaphase I of individual from Guaribas population; (d) metaphase II of specimen from Maracaípe population. Note the localization and the size of the signals between populations. Bar = 5 μm.

Mapping of *Dgmar*MITE probes on the karyotype of *D.* (*L.*) *gilletti* revealed signals in all chromosomes, except for pairs five and seven of the Igarassu population (Figure 3a), and pair five of the Aldeia population (Figure 3b). Overall, *Dgmar*MITE sequences were predominantly located in euchromatic regions in individuals from both populations, except in the Igarassu population, for which signals were detected at heterochromatic regions of chromosome pairs six and eight (Figure 3a). Similarly, in Aldeia population, *Dgmar*MITE was restricted to the heterochromatic region of pair two (Figure 3b). In addition, five heteromorphic pairs were observed in Aldeia individuals (Figure 3b).

Mapping of *Dgmar*MITE probes on the karyotype of *D.* (*L.*) *guaribensis* revealed their location in heterochromatic regions of all autosomes and of the X chromosome in both populations (Figure 3c,d). Additional signal was observed on the y chromosome of the Guaribas population (Figure 3c). Furthermore, *Dgmar*MITE sites were observed in euchromatic regions of all autosomes, except pair eight, in specimens from Maracaípe (Figure 3d). Overall, stronger FISH signals were observed in the karyotypes of individuals from Guaribas when compared to specimens from Maracaípe (Figure 3c,d). 

## Discussion

The karyotype observed in *D.* (*L.*) *guaribensis* (2*n* = 18) is considered derived from the ancestral number reported for the family Scarabaeidae (2*n* = 20), but conserved in most species of *Dichotomius*. The configuration of the sexual bivalent (Xy_r_), which has been reported so far only in *D. schiffleri*, also differs from the ancestral Scarabeidae Xy_p_ (Cabral-de-Mello *et al.*, 2008, 2011a; Silva *et al.*, 2009; Xavier *et al.*, 2014). The derived karyotypes of *D. gilletti* and *D. iannuzziae* observed in this study are similar to those described by Cabral-de Mello *et al.* (2011a) prior to the taxonomic revision by Valois *et al.* (2017). In Cabral-de Mello *et al.* (2011a), these species were referred to as *D. sericeus* and *D. laevicollis*, respectively. The presence of constitutive heterochromatic blocks in pericentromeric regions of all autosomes, as observed in *D.* (*L.*) *gilletti* and *D.* (*L.*) *guaribensis*, is a common feature in the genus *Dichotomius* (Cabral-de-Mello *et al.*, 2011c), and has also been reported for the other two species investigated herein: *D.* (*L.*) *iannuzziae* (Cabral-de-Mello *et al.*, 2011c) and *D.* (*L.*) *schiffleri* (Xavier *et al.*, 2014).

*Dgmar*MITE, the TE characterized here, presented features shared by all MITEs such as a large copy number, which was observable with FISH resolution, lack of transposase coding, AT richness, and conservation of TIR structure (Kuang *et al.*, 2009). In most cases, sequence similarity between a MITE and its closest element is restricted to the TIRs (Feschotte and Pritham, 2007). The similarity between the TIRs of *Dgmar*MITE and the *AfMar*2 element of *A. flavolineata* indicates that the former belongs to the *Mariner* family.

The presence of *Dgmar*MITE in the taxonomically similar species *D.* (*L.*) *gilletti* and *D.* (*L.*) *guaribensis* [considering the genus revision of Valois *et al.* (2017)] and therefore possibly phylogenetically closer, suggests an origin of this element in the common ancestor of these species. An alternative hypothesis is that this element may have originated independently in the species *D.* (*L.*) *gilletti* and *D.* (*L.*) *guaribensis* by horizontal transfer events, which are frequently observed for *Mariner*-like elements (Robertson, 1995; Robertson and Lampe, 1995), including MITEs, as described previously for the Stowaway element in the plant family Pooideae (Minaya *et al.*, 2013). The *Dgmar*MITE origin may be recent or not however, since older TEs accumulate preferentially in heterochromatic regions (Junakovic *et al.*, 1998), as has been previously proposed for other TEs in Scarabaeidae (Oliveira *et al.*, 2013). Genome colonization of this MITE possibly occurred earlier in *D.* (*L.*) *guaribensis* than in *D.* (*L.*) *gilletti.* In *D.* (*L.*) *gilletti*, the preferential location in euchromatic regions, the presence of heteromorphic pairs in the population of Aldeia, and absence of the signal in one or two chromosome pairs, suggest that this element originated most recently.

In addition to *D.* (*L.*) *gilletti*, predominantly euchromatic signals have been reported for TEs in *D.* (*L.*) *schiffleri* (Xavier *et al.*, 2014), for the grasshopper species *Eyprepocnemis plorans* (Montiel *et al.*, 2012) and *A. flavolineata* (Palacios-Gimenez *et al.*, 2014). The occurrence of *Dgmar*MITE in euchromatic regions can influence gene expression and/or gene and chromosome mutations (Kidwell and Lish, 2000; Feschotte and Pritham, 2007). However, it is also possible that the element is inserted in pseudogenes or even other dispersed repetitive sequences in euchromatin, as proposed for *Mariner* family elements of *E. plorans* (Montiel *et al.*, 2012). On the other hand, the presence of MITEs in heterochromatic regions, as observed for *Dgmar*MITE in *D.* (*L.*) *guaribensis*, is not common, since these TEs are preferentially associated with genes (Lu *et al.*, 2011). However, heterochromatic enrichment of these elements has been described in other organisms, such as in the insect *Anopheles gambiae* (Quesneville *et al.*, 2006) and in the plants *Oryza sativa* (Lu *et al.*, 2011) and *Arabidopsis thaliana* (Guo *et al.*, 2017).

Mapping of *Dgmar*MITE in *D.* (*L.*) *gilletti* and *D.* (*L.*) *guaribensis* showed variation in the location and number of sites between species and populations. These findings suggest that this non-autonomous element may be cross-mobilized to different regions of host genomes using the transposase of either an older or a newly emerged transposon, in this latter case *Dgmar*MITE accumulation occurs by a process known as “snowball effect” (Feschotte *et al.*, 2005). The transposase used by *Dgmar*MITE could belong to a, closely related TE as observed between the inactive *peach* element and the transposase of *Mariner*-like *Mos1* in *Drosophila melanogaster* (Garza *et al.*, 1991), or to a phylogenetically distant TE, as observed between an element of the Stowaway family and *Osmar* transposase, an autonomous *Mariner*-like element in the rice genome (Feschotte *et al.*, 2003; Yang *et al.*, 2009).

With respect to copy-number variation in heterochromatic regions of *D.* (*L.*) *guaribensis* chromosomes, an increase in *Dgmar*MITE copy number in the Guaribas population probably results from transposition-independent events, including ectopic recombination and concerted evolution. The latter has been observed for highly repetitive DNA sequences such as *Mariner* elements found in the heterochromatin of *Drosophila erecta* (Lohe *et al.*, 1995). An alternative hypothesis to explain this variation is that this element is undergoing a reverse process with quantitative and random copy loss in the genomes of individuals from Maracaípe population. In this scenario, *Dgmar*MITE would be undergoing senescence, the last stage of the transposable element “life cycle”, as described by Kidwell and Lisch (2000).

Mapping of *Dgmar*MITE in species of the *Dichotomius* (*Luederwaldtinia*) *sericeus* complex contributed to increase our knowledge about the location and distribution of TEs in dung beetle genomes. This analysis also revealed the accumulation of *Dgmar*MITE in the karyotype of two species. Plausible mechanisms underlying such accumulation include the occurrence of cross-mobilization and/or ectopic recombination in heterochromatic regions. However, we cannot completely rule out the possible involvement of other molecular mechanisms discussed here. Therefore, further characterization and chromosome mapping should be extended to other species within this complex of species.

## Supplementary material

The following online material is available for this article:

Figure S1 - Amplification of *Dgmar*MITE in four phylogenetically related species of the *Dichotomius* (*Luederwaldtinia*) *sericeus* complex.
