# Supplementary material for: Characterization and chromosomal mapping of the DgmarMITE transposon in populations of Dichotomius (Luederwaldtinia) sericeus species complex (Coleoptera: Scarabaeidae)
Source: Genet Mol Biol. 2018 Jun 4;41(2):419–25. doi: 10.1590/1678-4685-GMB-2017-0230 (PMC6082228; doi:10.1590/1678-4685-GMB-2017-0230)
Supplement: Supplementary file 1 [file 1415-4757-GMB-1678-4685-GMB-2017-0230-s001.pdf]

Supplementary Material to "Characterization and chromosomal mapping of the *Dgmar*MITE transposon in populations of *Dichotomius* (*Luederwaldtinia*) *sericeus* species complex (Coleoptera: Scarabaeidae)"

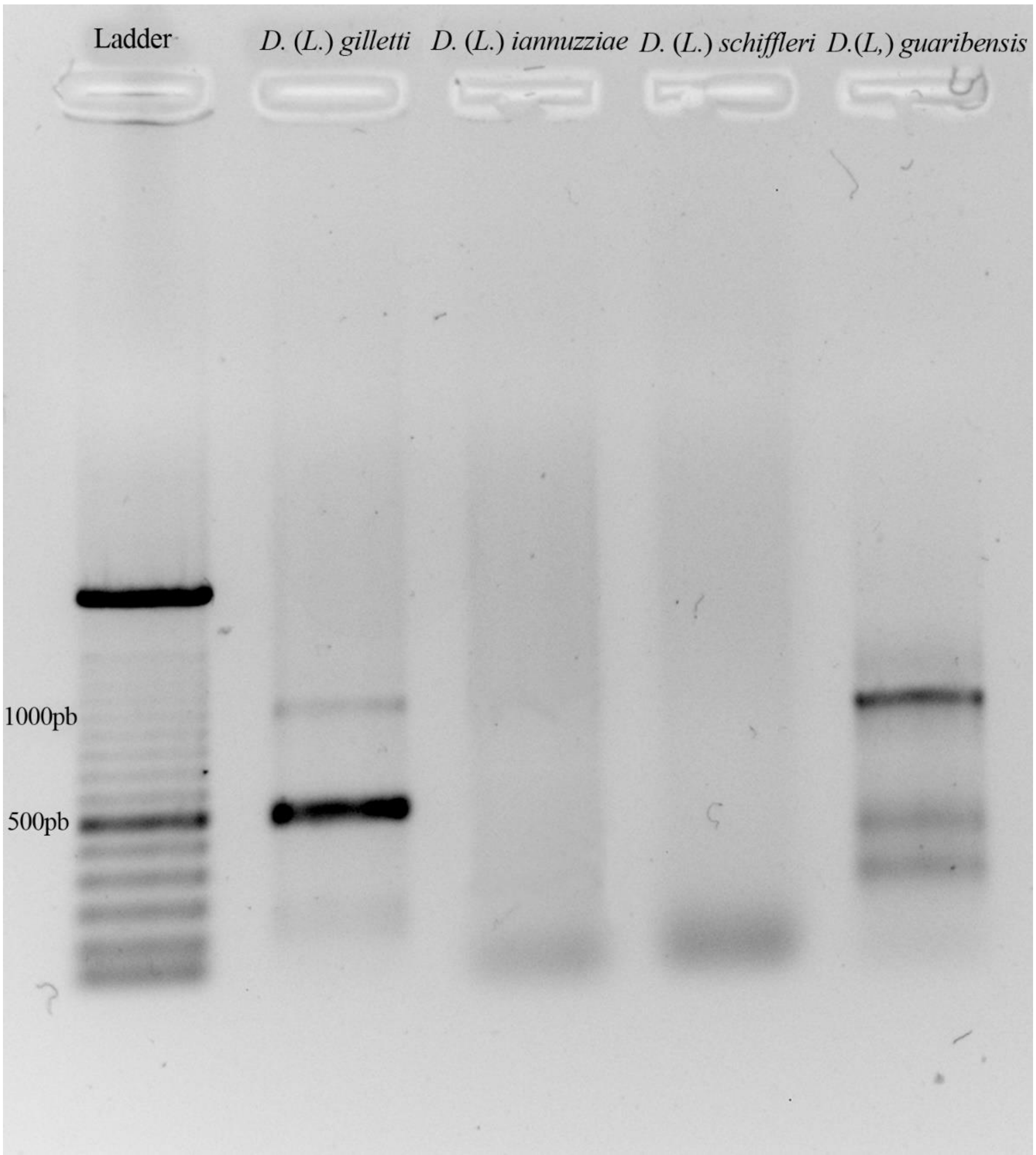

Figure S1 – Amplification of *Dgmar*MITE in four phylogenetically related species of the *Dichotomius* (*Luederwaldtinia*) *sericeus* complex.
